# Supplementary material for: Biotin-operated ligand trapping enables dynamic spatial redistribution of protein and organelle in living cells
Source: bioRxiv. 2026 Oct 6:2026.10.02.756291. Preprint. [Version 1] doi: 10.64898/2026.10.02.756291 (PMC13692045; doi:10.64898/2026.10.02.756291)
Supplement: Supplementary file 2 [file media-3.docx]

**Table S3.** Sequences of gRNA and primers used for knockin and validation of TP53-HaloTag-V5 cell line.

| Oligos | Sequence (5’ to 3’) |
| --- | --- |
| TP53 gRNA sequence | GGAGAATGTCAGTCTGAGTC |
| TP53 Junction primer | TATTTGCATGGGGTGTGGCC |
| HaloTag Junction primer | ATGATGTTGCGCCACACGTAGG |
| HaloTag Sanger primer | CTCGCCCAGGACTTCCACAT |
